# *Salmonella typhi* and Pregnancy: A Case Report

**DOI:** 10.1155/S1064744994000633

**Published:** 1994

**Authors:** Brion Gluck, Kirk D. Ramin, Susan M. Ramin

**Affiliations:** Department of Obstetrics and Gynecology University of Texas Southwestern Medical Center 5323 Harry Hines Boulevard Dallas TX 75235-9032. USA

## Abstract

*Background: Salmonella typhi* may be a cause of significant morbidity and mortality in both the mother and fetus. Febrile illness during pregnancy, especially that associated with hemolysis, is associated with chorioamnionitis, pyelonephritis, or viral syndrome. As such, *S. typhi* should be considered when a patient presents with a fever and hemolysis. We present a case of *S. typhi* complicating pregnancy.

*Case:* A primigravida at 26 weeks gestation presented with persistent spiking temperature and severe hemolysis. Her presenting signs and symptoms included fever, vomiting, cough, earache, jaundice, dark urine, and anemia. After 4 units of blood, her posttransfusion hematocrit was 29%. Hemolysis was evident on a peripheral blood smear. Total bilirubin was 2.5 mg/dl and direct bilirubin was 1.2 mg/dl. Gentamicin and clindamycin were administered empirically. Stool, blood, and urine cultures were obtained. Blood cultures were positive for *S. typhi*. Antibiotic coverage was changed to gentamicin and ceftriaxone. She defervesced on the 5th day and had no further problems. A healthy male infant was delivered vaginally at term.

*Conclusion:* Typhoid fever is a serious infection, and special concern arises when *S. typhi* complicates pregnancy. The diagnosis of *S. typhi* should be considered in a gravida presenting with fever with prompt institution of antibiotic therapy. Appropriate cultures are essential for confirming the diagnosis.


Infectious Diseases in Obstetrics and Gynecology 2:186–189 (1994)
© 1994 Wiley-Liss, Inc.

### *Salmonella typhi* and Pregnancy: A Case Report

**Brion Gluck, Kirk D. Ramin, and Susan M. Ramin**
*Department of Obstetrics and Gynecology, University of Texas Southwestern Medical Center, Dallas, TX*

Abstract

***Background:*** **Salmonella typhi** **may be a cause of significant morbidity and mortality in both the mother and fetus. Febrile illness during pregnancy, especially that associated with hemolysis, is associated with chorioamnionitis, pyelonephritis, or viral syndrome. As such, *S. typhi* should be considered when a patient presents with a fever and hemolysis. We present a case of *S. typhi* complicating pregnancy.**

***Case:*** **A primigravida at 26 weeks gestation presented with persistent spiking temperature and severe hemolysis. Her presenting signs and symptoms included fever, vomiting, cough, earache, jaundice, dark urine, and anemia. After 4 units of blood, her posttransfusion hematocrit was 29%. Hemolysis was evident on a peripheral blood smear. Total bilirubin was 2.5 mg/dl and direct bilirubin was 1.2 mg/dl. Gentamicin and clindamycin were administered empirically. Stool, blood, and urine cultures were obtained. Blood cultures were positive for *S. typhi.* Antibiotic coverage was changed to gentamicin and ceftriaxone. She defervesced on the 5th day and had no further problems. A healthy male infant was delivered vaginally at term.**

***Conclusion:*** **Typhoid fever is a serious infection, and special concern arises when *S. typhi* complicates pregnancy. The diagnosis of *S. typhi* should be considered in a gravida presenting with fever with prompt institution of antibiotic therapy. Appropriate cultures are essential for confirming the diagnosis.** © 1994 Wiley-Liss, Inc.

Key words
Hemolysis, typhoid fever, microbiologic clues

In 1901, Dr. Frank W. Lynch[1] reported 1,079 cases of typhoid fever at the Johns Hopkins Hospital. Two hundred eighty-nine of these cases were females but only 5 occurred during pregnancy. Subsequently, Hicks and French[2] in 1905 reported a 65–85% abortion or premature labor rate when *Salmonella typhi* infection complicated pregnancy. With the advent of antibiotics as well as improved sanitation, food processing, and storage, the incidence of typhoid fever in the United States has declined. Despite this decline, typhoid fever remains a serious complication in pregnant women. We present a case of *S. typhi* complicating pregnancy to emphasize the importance of an evaluation of geographic risks and microbiologic clues as well as hematologic sequelae.

### CASE REPORT

A 21-year-old Hispanic primigravida at 26 weeks gestation was transferred to our institution for persistent spiking temperature and hemolysis. She had been admitted to her local hospital with fever to 40°C, vomiting, cough, and an earache. During that admission, the patient was noted to have dark urine, anemia, and jaundice. A presumptive diagnosis of hepatitis was made and she received 4 units of blood. Seven days prior to admission, she was prescribed amoxicillin, even though she had a his-

**Address correspondence/reprint requests to Dr. Susan M. Ramin, Department of Obstetrics and Gynecology, University of Texas Southwestern Medical Center, 5323 Harry Hines Boulevard, Dallas, TX 75235-9032.**

Received June 23, 1994

Accepted September 23, 1994

tory of penicillin allergy. An obstetric abdominal ultrasound was consistent with a 26-week gestation, as well as a normal liver and gallbladder. The initial vital signs at transfer were a blood pressure of 100/70, pulse of 86, respiratory rate of 18, temperature of 37.8°C. She was jaundiced but had no petechiae or other skin lesions. Her chest was clear and she had no costovertebral angle tenderness. Her abdomen was soft without tenderness and no hepatosplenomegaly. Her fundal height was 25 cm with a soft nontender uterus. A bimanual examination revealed a long, closed cervix and intact membranes. On admission, her hematocrit was 29.3%, platelet count 187,000/mm$^3$, and white blood cell (WBC) count 11,500/mm$^3$ with a differential of 81% polymorphonuclear cells, 17% lymphocytes, 4% monocytes, and 1% basophils. Her peripheral smear revealed evidence of hemolysis even though it was obtained posttransfusion. A urinalysis was significant for a specific gravity of 1.010, pH of 7.0, bilirubin of 4.0, and urobilinogen of 1.2. The results of serum chemistry revealed a potassium of 3.3 mEq/l, glucose of 184 mg/dl, blood urea nitrogen of 6 mg/dl, and albumin of 2.8 g/dl. An evaluation of liver function revealed an aspartate aminotransferase of 69 U/l, alkaline phosphatase of 143 U/l, total bilirubin of 5.4 mg/dl, direct bilirubin of 1.8 mg/dl, and a $\gamma$-glutamyltranspeptidase of 72 U/l. Stool, urine, and blood cultures were obtained. Gentamicin and clindamycin were administered empirically with no laboratory evidence of further hemolysis. Blood cultures were subsequently positive for *Salmonella* species, despite the fact that the patient had received ampicillin. Antibiotic coverage was changed to gentamicin and ceftriaxone. She defervesced on the 5th hospital day and was discharged on hospital day 10 with a repeat hematocrit of 23.8%. Urine and stool cultures were sterile. The final report on the organism isolated from a blood culture was *S. typhi*. She delivered vaginally a 2,845 g male infant with an Apgar score of 9 at both 1 and 5 minutes at 38 weeks estimated gestational age. There were no further complications, and both mother and infant left the hospital in good condition.

### DISCUSSION

*S. typhi* is a facultative anaerobic gram-negative enteric rod that causes a wide variety of human infections when ingested in contaminated food or water. In addition to typhoid fever, focal systemic infections, septicemia, and gastroenteritis can occur.[3] The common presenting findings are summarized in Table 1. Infection with *S. typhi* is relatively rare and the incidence of infection in the United States has remained unchanged in recent years at 0.2/100,000 population.[3] *Salmonella* is most commonly acquired during travel to countries in which it is endemic, e.g., Mexico, Indian subcontinent, Southeast Asia, and Indonesia.[3] Alexandria, Egypt, Jakarta, Indonesia, and Santiago, Chile, are the leading geographic locations. The incubation period ranges from a few days to several months with associated headache, malaise, and chills. A recurrent febrile response is usually seen after the bacteria invade the mucosa of the small intestine which, in turn, causes a transient bacteremia. This is followed by continued multiplication of organisms within phagocytic cells and sustained episodes of secondary bacteremia.

**TABLE 1. Common presenting signs and symptoms of typhoid fever[3,4,6,9]**

| |
|---|
| Persistent fever |
| Abdominal pain |
| Diarrhea |
| Hypothermic response to antipyretics |
| Headache |
| Cough |
| Constipation |
| Splenomegaly |
| Rose spots (small petechial hemorrhages) |
| Generalized malaise |
| Anorexia |
| Chills |
| Relative bradycardia |
| Leukopenia |

Special concern arises when pregnancy is complicated by *S. typhi* (Table 2). Prior to the antibiotic era, maternal mortality was increased and the fetus was at risk for infection with subsequent abortion.[2] More recently, Riggall and associates[4] reported an improved prognosis for both mother and fetus in 7 cases of typhoid fever complicating pregnancy. The diagnosis was confirmed in all cases by blood culture. Therapy consisted of high doses of ampicillin for 2 weeks. All but 1 pregnancy resulted in good outcomes. One pregnancy ended in a spontaneous abortion.

Although typhoid fever is a rare disease in the United States, sporadic infections do occur. Ac-

**TABLE 2. Various adverse outcomes and morbidity associated with typhoid fever in pregnancy[1,2,4]**

| |
|---|
| Maternal mortality |
| Blood transfusions |
| Premature labor |
| Spontaneous abortion |
| Fetal infection |

**TABLE 3. Geographic areas of 203 reported cases of *S. typhi* in the United States from January 1, 1994, to July 23, 1994[5]**

| Reporting area | No. of cases |
|---|---|
| Pacific | 50 |
| Mid-Atlantic | 49 |
| East/North Central | 38 |
| South Atlantic | 33 |
| New England | 16 |
| West/South Central | 8 |
| Mountain | 7 |
| East/South Central | 2 |
| West/North Central | 0 |

cording to the Centers for Disease Control,[5] there have been 203 cases of typhoid fever reported in the United States from January 1, 1994, to July 23, 1994. The geographic areas from which cases of typhoid fever have been reported are summarized in Table 3. The states reporting the majority of the cases of typhoid fever include Massachusetts (N = 12), New York (N = 29), New Jersey (N = 14), Illinois (N = 18), Florida (N = 20), and California (N = 50). Moreover, typhoid fever is a serious infection when it does occur. The diagnosis of *S. typhi* is often not considered when a patient presents with fever. It is of paramount importance that the diagnosis in pregnancy be made with prompt institution of antibiotic therapy which is necessary for improved maternal and fetal prognosis.[6] Appropriate cultures obtained prior to the initiation of antibiotic therapy are essential for confirming the diagnosis. Blood cultures are positive early in the course of infection, while stool cultures are positive later in the course of the infection. Cultures of multiple sites (urine, amniotic fluid, skin scrapings, bone marrow) improve the yield of a positive culture.

The recommended treatment regimens for *S. typhi* in pregnancy are summarized in Table 4. Chloramphenicol continues to be the treatment of choice; however, ampicillin or trimethoprim/

**TABLE 4. Antimicrobial agents commonly used in the treatment of *S. typhi* in pregnancy[3,4,8]**

| |
|---|
| Ampicillin, 8 g/day IV |
| Chloramphenicol, 3–4 g/day orally for 14 days[a] |
| Ceftriaxone, 75 mg/kg/day for 5 days |
| Trimethoprim, 640 mg/day and sulfamethoxazole, 3,200 mg/day, orally in 2 divided doses[a] |
| Amoxicillin, 4–6 g/day in 4 divided doses |
| Cefoperazone, 2 g b.i.d. IV to 4 g q.i.d. |

[a]May be given IV if patient cannot tolerate oral medication.

sulfamethoxazole may also be given. Chloramphenicol readily crosses the placenta but has not been shown to be teratogenic when utilized in early pregnancy.[7] It is unlikely that "gray baby syndrome" (cyanosis, vascular collapse, and death), which has been reported when this antibiotic was given to the premature infant, would be caused by transplacental passage of this drug.[8] The fluoroquinolones, i.e., ciprofloxacin and ofloxacin, have been shown to be efficacious in the treatment for *S. typhi;* however, they are currently not recommended during pregnancy. Although they have not been shown to be teratogenic in humans, there are reports of irreversible arthropathy in immature animals. Thus, this class of drugs should be reserved for serious life-threatening infection for which other antibiotics are ineffective or inappropriate.

The present case had the clinical features of unrelenting spiking high fevers, severe hemolysis (for which blood transfusions were given), and obvious illness. She did not have diarrhea which may occur late in the course of the infection. We concur with Dildy and colleagues[9] that the clinician must also consider geographic risks as well as microbiologic and hematologic clues in order to establish the proper diagnosis.

### REFERENCES

1. Lynch F: Fetal transmission of typhoid. JAMA 36:1136, 1901.
2. Hicks HT, French H: Typhoid fever and pregnancy with special reference to foetal infection. Lancet 1:1491–1493, 1905.
3. Keusch GT: Salmonellosis. In Willson JD, Braunwald E, Isselbacher KJ, Petersdorf RG, Martin JB, Fauci AS, Root RK (eds): Harrison's Principles of Internal Medicine. 12th Ed. New York: McGraw-Hill, pp 609–611, 1991.
4. Riggall F, Salkind G, Spellacy W: Typhoid fever complicating pregnancy. Obstet Gynecol 44:117–121, 1974.

5. Centers for Disease Control: Cases of selected notifiable diseases, United States, weeks ending July 23, 1994 and July 24, 1993 (29th week). MMWR 43:540, 1994.
6. Seoud M, Saade G, Uwaydah M, Azoury R: Typhoid fever in pregnancy. Obstet Gynecol 71:711–714, 1988.
7. Heinonen OP, Slone D, Shapiro S: Birth Defects and Drugs in Pregnancy. Littleton, MA: Littleton Publishing Sciences Group, 1977.
8. Gant NF: Drugs and medication during pregnancy. In: Cunningham FG, MacDonald PC, Gant NF, Leveno KJ, Gilstrap LC (eds). Williams Obstetrics, 19th ed. Norwalk, CT: Appleton & Lange, p 962, 1993.
9. Dildy GA III, Martens MG, Faro S, Lee W: Typhoid fever in pregnancy: A case report. J Reprod Med 35:273–276, 1990.
